# Community-based participatory research projects and policy engagement to protect environmental health on St Lawrence Island, Alaska

**DOI:** 10.3402/ijch.v72i0.21656

**Published:** 2013-08-05

**Authors:** Pamela K. Miller, Viola Waghiyi, Gretchen Welfinger-Smith, Samuel Carter Byrne, Jane Kava, Jesse Gologergen, Lorraine Eckstein, Ronald Scrudato, Jeff Chiarenzelli, David O. Carpenter, Samarys Seguinot-Medina

**Affiliations:** 1 Alaska Community Action on Toxics, Anchorage, AK, USA; 2 Native Village of Savoonga, St Lawrence Island, AK, USA; 3 Institute for Health and the Environment, University at Albany, Albany, NY, USA; 4 Department of Geology, St Lawrence University, Canton, NY, USA

**Keywords:** community-based participatory research, Yupik, persistent organic pollutants, polychlorinated biphenyls, environmental health, military toxics

## Abstract

**Objectives:**

This article synthesizes discussion of collaborative research results, interventions and policy engagement for St Lawrence Island (SLI), Alaska, during the years 2000–2012.

**Methods:**

As part of on-going community-based participatory research (CBPR) studies on SLI, 5 discrete exposure-assessment projects were conducted: (a) a biomonitoring study of human blood serum; (b–d) 3 investigations of levels of contaminants in environmental media at an abandoned military site at Northeast Cape – using sediment cores and plants, semi-permeable membrane devices and blackfish, respectively; and (e) a study of traditional foods.

**Results:**

Blood serum in residents of SLI showed elevated levels of polychlorinated biphenyls (PCBs) with higher levels among those exposed to the military site at Northeast Cape, an important traditional subsistence-use area. Environmental studies at the military site demonstrated that the site is a continuing source of PCBs to a major watershed, and that clean-up operations at the military site generated PCB-contaminated dust on plants in the region. Important traditional foods eaten by the people of SLI showed elevated concentrations of PCBs, which are primarily derived from the long-range transport of persistent pollutants that are transported by atmospheric and marine currents from more southerly latitudes to the north.

**Interventions:**

An important task for all CBPR projects is to conduct intervention strategies as needed in response to research results. Because of the findings of the CBPR projects on SLI, the CBPR team and the people of the Island are actively engaging in interventions to ensure cleanup of the formerly used military sites; reform chemicals policy on a national level; and eliminate persistent pollutants internationally. The goal is to make the Island and other northern/Arctic communities safe for themselves and future generations.

**Conclusions:**

As part of the CBPR projects conducted from 2000 to 2012, a series of exposure assessments demonstrate that the leaders of SLI have reason to be concerned about the health of people due to the presence of carcinogenic chemicals as measured in biomonitoring and environmental samples and important traditional foods.

Levels of polychlorinated biphenyls (PCBs) are declining in most of the developed countries since the manufacture and use of these substances has been curtailed. However, the Polar Regions are reservoirs for atmospheric transportation of persistent toxic substances, which are carried by air currents and then condense out of the vapour phase in the cold of the Polar Regions. This fact, plus the high-fat diet of many Indigenous communities in the Arctic, raises concern that the intake of organochlorine compounds, which include PCBs, dioxins/furans and persistent organochlorine pesticides, may pose serious health risks to these populations (1).

St Lawrence Island (SLI), the largest island in the Bering Sea, is a mountainous landscape of volcanic rock and tundra that lies 240 km south of the Arctic Circle. The Island is about 140 km long and 13–36 km wide. It is situated only 61 km from the Chukotka Peninsula of northern Russia, closer than to the Alaska mainland at Nome which lies more than 322 km to the east of the community of Gambell (Fig. 1). The population of SLI consists of about 1,400 Yupik people living in 2 villages, Gambell and Savoonga. The SLI Yupik follow a traditional life style, which includes consumption of marine mammals and fish, bird eggs, as well as local greens and berries. Storage of meats is usually by drying in the brief summer, freezing or fermenting in pits in the ground (2).

**Fig. 1:**
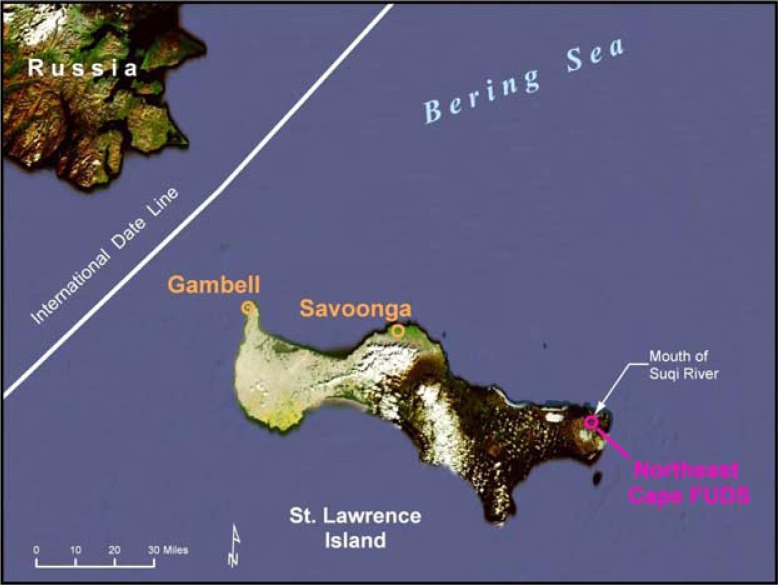
Map of St Lawrence Island, Alaska, showing both villages and the formerly used defense site (FUDS) at Northeast Cape.

Two US military bases were constructed on SLI because of its strategic location. From 1948 to 1956, the US Air Force operated an aircraft control and warning station at Gambell, but moved the operation in 1952 to Northeast Cape where a large military installation was built and operated until 1972. Gambell is built on coarse gravel, and fuels, oils, and other hydrocarbons that spilled have settled in a layer above the permafrost, some 8–10 feet below the surface. There is no documented history of the use of PCBs at Gambell, although some use is likely in generators, transformers and paint.

The other military base was located at the opposite end of the island, at Northeast Cape during the Cold War. While there is currently no permanent settlement at Northeast Cape, the region has a number of hunting and fishing camps where members of some Savoonga families spend several months to hunt seal, walrus and whale, as well as to catch fish from the local rivers and streams and near shore areas and collect berries and greens. When the military base at Northeast Cape was closed in 1972, most of the supplies and materials present at the site, including buildings, heavy equipment and generating facilities, as well as fuel containers, were abandoned or buried on-site (1, 3).

In addition to abandoned buildings, radar stations and air strips, the military left a range of contaminants, including fuels, PCBs, solvents, pesticides and various trace metals, which had a severe impact on the local environment. The military complex was located along the Bering Sea. The soils are typical of tundra-covered areas, with numerous wetlands, ephemeral ponds and small streams draining from the granitic mountains. There was a documented diesel fuel spill of 160,000 gallons in the late 1960s that killed fish and other animals in the Suqitughneq (Suqi) River. The area around the power-generating station and the soils and plants down-gradient of the main complex are highly contaminated with PCBs and volatile organics, as determined as a part of the on-going remediation of the site under the direction of the US Army Corps of Engineers and their contractors. While a multimillion dollar remediation of the site at Northeast Cape is currently being conducted by the Corps, contamination remains.

The perception in the Yupik community is that, in the recent years, there has been an increase in the levels of various diseases, especially cancer, and that this has occurred particularly among those individuals who have spent significant time at Northeast Cape (4). The concern in the community is that these increases in disease have resulted from environmental contamination originating from the former military sites. One woman, Annie Alowa, a community health aide for Savoonga requested technical assistance from Alaska Community Action on Toxics (ACAT), an environmental health research and advocacy organization based in Anchorage. This contact ultimately resulted in a series of federal grants to ACAT and SLI for community-based participatory research (CBPR) projects. CBPR is “a methodology that promotes active community involvement in the processes that shape research and intervention strategies, as well as in the conduct of research studies” (5). Suk et al. note the need to “assess biomarkers of exposure susceptibility and monitor the diet and health of native Arctic populations within the context of carefully designed community-based participatory research programs” (6). These CBPR projects are collaborations with the communities of SLI and universities, and are facilitated by ACAT.

The residents of SLI have at least 2 possible sources of exposure to environmental contaminants local and distant. The local source is from 2 abandoned military sites which are in areas of human activity and food collection. In addition, many organochlorines, including PCBs, dioxins/furans and persistent pesticides, travel from distant sources to concentrate in the polar regions by vapour-phase transport followed by precipitation in the cold climate (global distillation) or by ocean currents. These lipophilic compounds then bioconcentrate in the food chain and are ultimately consumed by humans (7, 8). Native Yupik people, whose diet includes the significant consumption of marine mammal fats, are susceptible to major exposure via this route.

At the request of the community, 5 CBPR exposure-assessment projects were conducted with SLI since 2000. We assessed contamination by (a) monitoring human blood serum, (b) analyzing sediment cores and plants, (c) using semi-permeable membrane devices (SPMDs), (d) collecting blackfish and (e) examining prepared traditional foods. These CBPR projects support community-led interventions aimed to achieve policies that better protect the health and environment of the Yupik people of SLI. This article synthesizes discussion of collaborative research results, interventions and policy engagement for SLI during the years 2000–2012.

## Summaries of 5 CBPR exposure assessments

### Human blood serum from residents of SLI

In 2001–2003 a biomonitoring project was conducted on SLI by the CBPR team. Our methods and results are more fully described in the study by Carpenter et al. (1). Our work demonstrated that the Yupik people of SLI have much higher body burdens of PCBs than populations in the lower-48 states and Canada, suggesting that the long-range transport to this northern region underlies the elevated PCB blood serum levels found in the people of both Gambell and Savoonga. Additionally, the considerably higher levels among those Savoonga residents associated with Northeast Cape suggest added exposures to contamination from the military site. The research team found that the people of SLI who had close familial ties to Northeast Cape had the highest mean lipid-adjusted levels of PCBs in blood serum (1,143 ppb) compared with other residents of Savoonga (847 ppb) and Gambell (785 ppb).

### Sediment cores and plants at Northeast Cape

In 2002, 2006 and 2007, the CBPR team conducted sediment core and plant sampling at Northeast Cape and other sites to determine if contaminants are derived from military sources or from long-range transport. Our methods and results are more fully described in the study by Scrudato et al. (9). We collected plant samples and sediment cores at military and remote control sites on SLI and mainland Norton Sound and analyzed the samples for PCB congeners, mirex (a pesticide), dichlorodiphenyldichloroethylene (DDE – a metabolite of the pesticide dichlorodiphenyltrichloroethane (DDT)), and hexachlorobenzene (HCB) (pesticide and industrial by-product of chemical manufacturing).

Our work showed that plants collected in the vicinity of the main operation complex at Northeast Cape had the highest concentrations of PCBs, and it was determined that most of the contamination was derived from PCB-contaminated dust that was redistributed onto the plants from cleanup activities at the time. We discussed the findings with the leaders of SLI, who asked us to present the information at community meetings. The people concurred that it was not advisable to harvest edible plants or berries from Northeast Cape in the foreseeable future in order to prevent exposure to PCBs. If plants are to be harvested, they should be thoroughly rinsed with clean water. Congener patterns, overall concentrations of PCBs and proximity to the main operation complex compared with other sites clearly indicated the military site as the primary source of contamination of the plants at Northeast Cape.

The purpose of collecting sediment cores in the Suqi River and estuary was to determine deposition and congener patterns over time in order to discern predominance of military sources or long-range transport. The research team found that the river is a source of PCBs, as there are areas in the upstream reaches of the river's drainage from the formerly used defence site (FUDS) that serve as a continuing source of PCBs to the Suqi watershed.

### Semi-permeable membrane devices in Suqi River watershed

#### Method

In the summers of 2007 and 2008, the research team deployed SPMDs in several locations within the Suqi River and its estuary at Northeast Cape downstream from the main operation centre of the FUDS. SPMDs are sophisticated contaminant samplers developed by the US Geological Survey, and are designed to measure concentrations of persistent organic pollutants (POPs) in the water column integrated over time. They have been described as “virtual fish” because they assess biologically relevant concentrations of POPs (8, 10). The SPMDs were deployed for 28 days and dialysates analyzed for POPs (9, 11).

#### Preliminary results

**Fig. 2:**
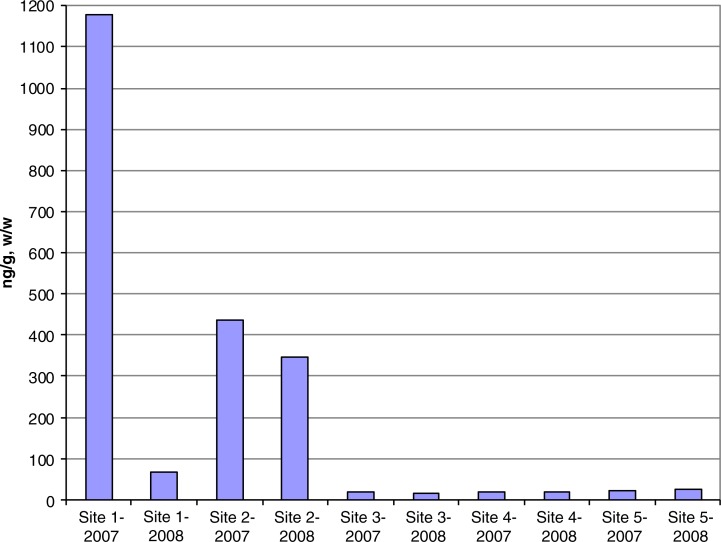
Comparison of contaminant levels at 5 sites in SPMDs from the Suqi River on St Lawrence Island, Alaska, 2007 and 2008.

**Fig. 3:**
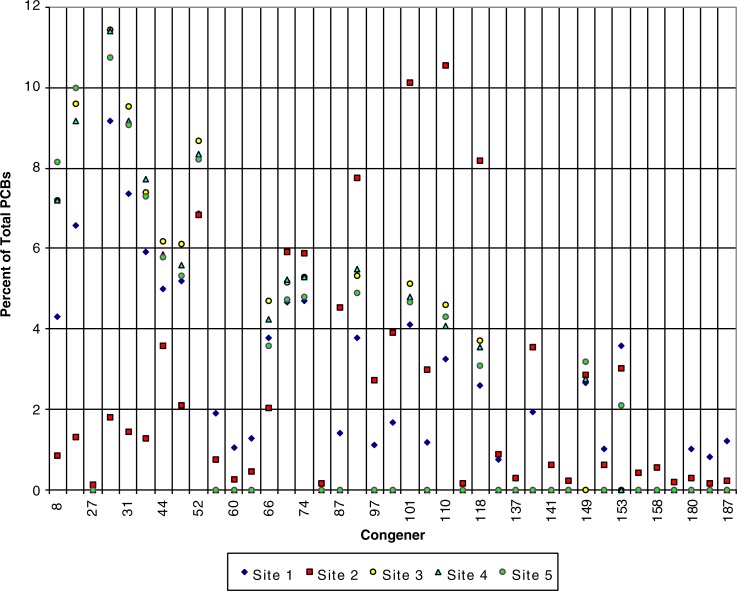
Percent of total PCBs by congener in SPMD samples for 2007 from the Suqi River, St Lawrence Island, Alaska.

**Fig. 4:**
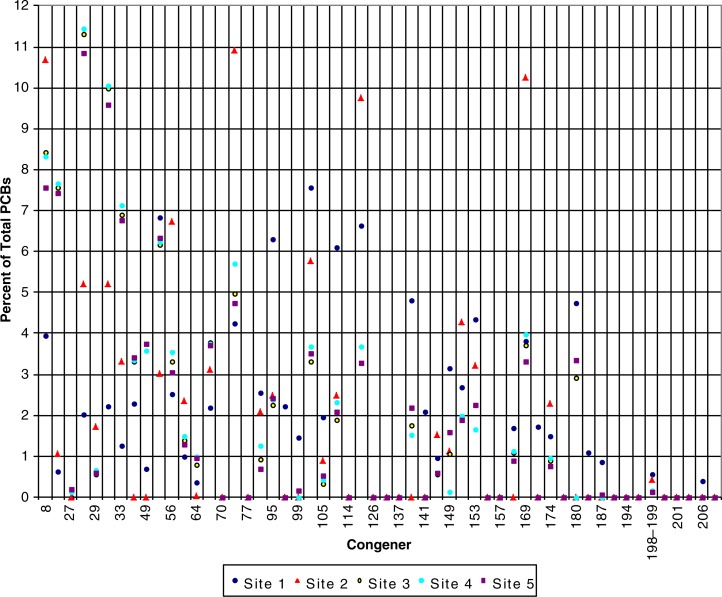
Percent of total PCBs by congener in SPMD samples for 2008, from the Suqi River, St Lawrence Island, Alaska.

Preliminary analyses of SPMD samples show PCB levels ranging from a maximum of 1,180 ng/g in 2007 and 347 ng/g in 2008 (Fig. 2). In the 2007 samples, substantially higher levels of PCBs were found just downstream from the main operation centre at the Northeast Cape FUDS with decreasing concentrations downstream, ranging from the highest concentration of 1,180–17 ng/g. The sample concentrations from 2008 ranged from 347 ng/g at an upstream site to 15 ng/g further downstream, with the highest concentration found at the site slightly more distant from the main operation centre than the closest sampling location. The lower chlorinated congeners composed a higher percentage of total PCBs both in 2007 and 2008 (Figs. 3 and 4).

#### Discussion

**Fig. 5:**
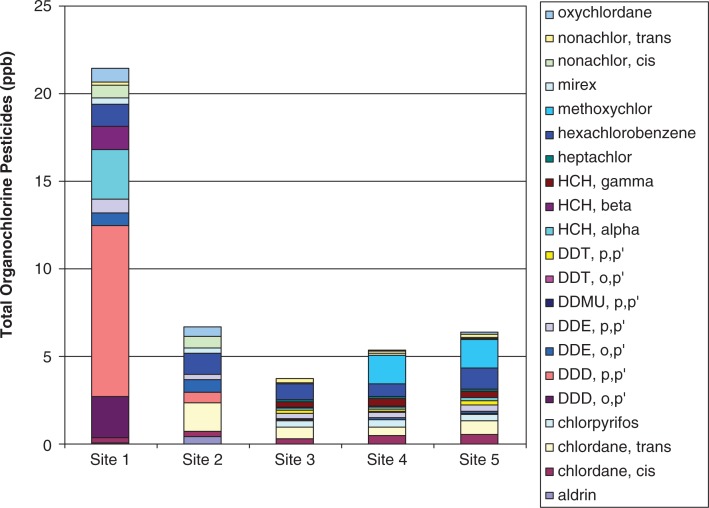
Organochlorine pesticide levels at 5 sites in SPMD samples for 2007 from the Suqi River, St Lawrence Island, Alaska.

**Fig. 6:**
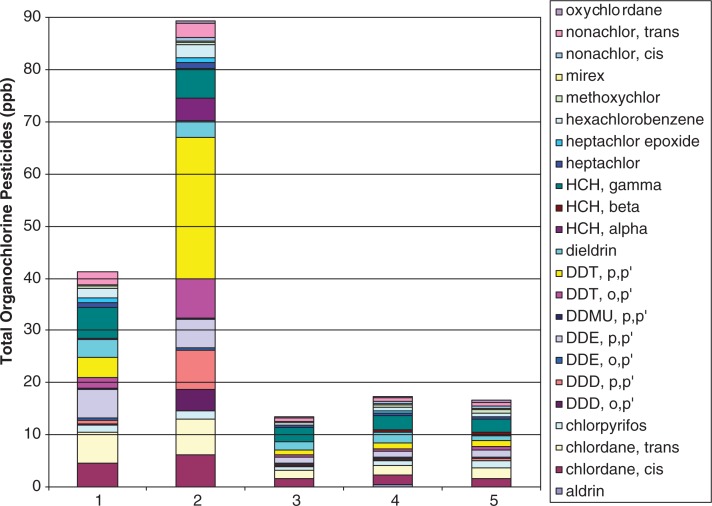
Organochlorine pesticide levels in 5 sites in SPMD samples for 2008 from the Suqi River, St Lawrence Island, Alaska.

Such high levels of PCBs and the dominance of lower chlorinated congeners indicate continuing contamination of the Suqi River from an upstream source. Higher chlorinated congeners are less water soluble and more persistent in the environment (12, 13). The pesticides with the highest concentrations in the SPMD samples were DDT and degradates, chlordane and degradates, HCB and HCH (Figs. 5 and 6). These preliminary results support the theory of continuing exposure from the FUDS which is upgradient from the deployed SPMDs.

A comparison of the current data to other SPMD studies suggests that there are much higher concentrations of bioavailable PCBs and organochlorine pesticides at Northeast Cape than at other locations. Concentrations of PCBs and organochlorine pesticides measured in SPMDs at the Three Gorges Reservoir on the Yangtze River, China – one of the most polluted rivers in the world – were lower than those in this study (14). An SPMD study of PCB levels was conducted in a contaminated Swedish bay that found concentrations ranging from 270 ng/g PCB within the bay to significantly lower concentrations of 3.6 ng/g at a control site outside the bay (15). Most notably, PCB concentrations in SLI are higher than those found in other regions of Alaska (9). Short et al. conducted a series of SPMD studies in multiple aquatic environments within salmon streams, intertidal environments, boat harbours and anchorages within Prince William Sound. The highest PCB concentration measured was 41.8 ng/g. Excluding the 3 most contaminated sites, 90% of PCB concentrations were below 7 ng/g. By comparison, even the lowest concentrations in the Suqi River on SLI were approximately 7 ng/g, while most of the Suqi River values exceeded the highest concentration found in Prince William Sound (11).

### Blackfish in the Suqi River

#### Method

In 2008, the CBPR team collected 5 blackfish (*Dallia pectoralis*) samples from the Suqi River and analyzed for 55 PCB congeners. Higher trophic level fish are not readily available in the Suqi River because the river and its fish populations have not recovered from damage caused by the military site.

#### Preliminary results

**Fig. 7:**
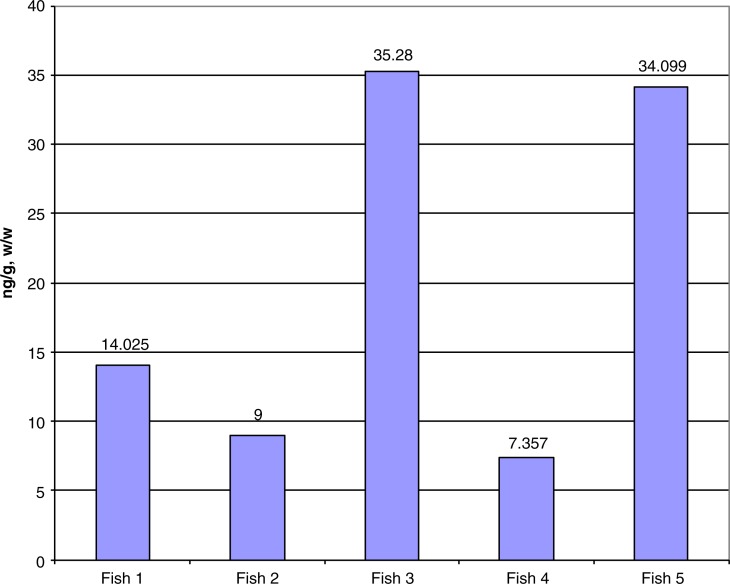
Concentrations of PCBs in 5 blackfish samples from the Suqi River, St Lawrence Island, Alaska.

PCB levels ranged from approximately 7 to 35 ng/g w/w (wet weight) in whole fish (Fig. 7). Blackfish samples from the Suqi River on SLI show a prevalence of the heavier PCB congeners, indicating military rather than global sources, as lighter congeners are carried by air currents and heavier congeners denote local sources of contamination, for example generators brought to the tundra by the military.

#### Discussion

Blackfish are not used as a subsistence resource, but the US EPA recommends limits on eating fish with PCB levels in this range (16). A previous health consultation conducted by the Agency for Toxic Substances and Disease Registry found an average PCB level of 100 ng/g, w/w in blackfish from the Suqi River (17). For comparison, in 2004, the State of Alaska conducted a study of multiple fish species in Alaska and found PCB levels that ranged from 0.94 ng/g w/w in pollock to 12.57 ng/g w/w in sockeye salmon (18). When compared to these data, blackfish from the Suqi River appear to have higher levels of PCBs relative to other fish in Alaska. A study of PCB contamination from a radar site in Labrador (in northern Canada) found higher levels of PCBs in sculpin – an average level of 800 ng/g w/w (19).

### Contaminants in traditional foods at SLI

At the request of the people on SLI, Yupik community field researchers on SLI began collecting samples in 2005 from a wide range of fresh and prepared traditional foods to determine food safety. They shipped them to our SLI Yupik staff in Anchorage, who checked the packing and sent the samples on to labs at the University at Albany, Institute for Health and the Environment. Our methods and results are more fully described in the study by Welfinger-Smith et al. (20).

Four years of data indicate high levels of PCBs in the rendered oils and blubber. The results for the PCB analysis show that many of the rendered oil and bowhead blubber samples exceeded 300 (ng/g, w/w); and bearded and spotted seal, polar bear and bowhead whale mungtak samples (blubber and skin delicacies) exceeded 100 ng/g, w/w on average.

As a frame of reference, the US EPA published a risk-based consumption limit for PCBs in fish of 1.5 (ng/g, w/w) for unlimited consumption so as to avoid excess risk of cancer (14). Most meat samples and greens from our SLI study were near this value, with the exception of polar bear and glaucous gull muscle tissues that were considerably higher. The results indicate plants and muscle tissue of meat for most species to be lowest in PCB concentrations.

High levels of PCBs were also found in blubber samples of 91 seals taken from the region at Point Hope, Diomede and Hooper Bay (21). Another study found that the mean PCB level of blubber samples taken from 20 bowhead whales near Barrow, Alaska was 458.5 (ng/g, w/w) (22). A study conducted to assess dietary exposures to POPs and metals in the nearby Chukotka region of the Russian Arctic found that consumption limits were warranted for certain marine and freshwater fish, some muscle tissues of waterfowl and seal, blubber of whale and seal, liver of most animals tested, and kidney tissues of reindeer, walrus, and seal (23). Researchers found elevated levels of PCBs in marine fish near a formerly used military base at Adak Island in the Aleutians that would trigger strict fish consumption advisories according to US EPA risk-based guidance (24).

## CBPR intervention strategies

Throughout the years, each time we received results from the labs, the CBPR team reported to the SLI leaders about our biomonitoring, environmental sampling and traditional food projects. Together decisions were made about the best way to inform the people on the Island – usually through community meetings at each village. The people of SLI solve problems by consensus, and they usually decide to inform officials of state and federal agencies in order to garner any governmental assistance available to them. They want the general public to know, also, so others can be aware of environmental contaminants. An example is the presence of mirex at Northeast Cape discovered by our CBPR team which indicates military use of this chemical which is now banned. Mirex is not listed as chemicals to be analyzed as part of the site assessments of FUDS (25), so making our data available to the military and the public can prompt the Army Corps of Engineers to include mirex in site assessments of other FUDS throughout the United States.

There is consternation among the SLI people about the safety of their traditional foods (26, 27). They have opted to refrain from harvesting plants at Northeast Cape and to avoid drinking the water or harvesting fish from the Suqi River. Since the military occupation, the Suqi has been devoid of large fish eaten for subsistence by the people of SLI. However, they decided to continue eating their other traditional foods while also continuing to work with the CBPR team to prompt policymakers locally, nationally and internationally to address contaminants affecting the Arctic. They want to make it safer for future generations to live on SLI and eat traditional foods.

### Local intervention strategies

Because of these CBPR projects, the people of SLI are actively engaging in interventions to ensure the responsible cleanup of the abandoned military sites at Northeast Cape and Gambell. In 2001, the SLI community and ACAT prompted the US Army Corps of Engineers to establish a formal Restoration Advisory Board (RAB) as required under the US Department of Defense RAB Rule and guidance provisions of the Defense Environmental Restoration Program. The RAB provides a regular forum for community members to review, provide comments and advise the process of environmental restoration. We also worked to secure successive “Technical Assistance for Public Participation” grants beginning in 2001 for the community to have their own independent scientific advisor who participates in the RAB meetings. They chose Ronald J. Scrudato, geology faculty and director of New York University's Environmental Research Center at Oswego, New York. They knew Dr Scrudato, because he was serving as principal investigator for ACAT's first CBPR grant at SLI, and they kept him as RAB advisor each time the Technical Assistance position came up for renewal. Although the RAB is advisory, it has motivated the Army Corps of Engineers to provide information about their remedial investigation and cleanup activities that was not given to the communities before the existence of the RAB. Advocacy by the people of SLI and ACAT resulted in the US Army Corps of Engineers’ moving the SLI FUDS from a low priority to one of the highest priorities for clean-up in Alaska.

In 2009, a delegation of 16 community leaders from SLI, ACAT, and collaborating scientists travelled to Washington DC to offer a series of briefings for high-level federal officials at the Department of Defense, NIEHS, EPA, Department of State, and members of Congress. As a result of these community interventions, officials now recognize the need to address military toxics and long-range transport of contaminants into Alaska. The US EPA made a commitment to establish an on-going formal St Lawrence Island Dialogue Group composed of officials from state and federal agencies, SLI tribal governments, the Alaska Native Tribal Health Consortium, ACAT investigators and collaborating scientists from the ACAT research team. The Dialogue Group is working to resolve concerns of the community about the clean-up of the FUDS and also to address health concerns. For the Northeast Cape site alone, approximately $100.1 million has been spent on remedial investigation and removal of buildings, debris and hazardous materials; and $7.3 million on remediation of the Gambell FUDS. Additional funds were appropriated to the SLI tribes as part of the Native American Lands Environmental Mitigation Program for debris removal (28). Residents of SLI are not convinced that cleanup is close to completion and are working to ensure that the Army Corps of Engineers accomplishes responsible cleanup. They expect remediation of contaminants that remain in the subsurface and groundwater at both FUDS. In 2012, the Agency for Toxic Substances and Disease Registry made an unprecedented commitment to conduct a formal public health assessment that considers “all possible sources of exposure” including local military sources and the long-range transport of POPs to the Island (Agency for Toxic Substances and Disease Registry, personal communication, 22 February 2012).

In August 2011, ACAT received a 5-year CBPR grant from the NIEHS to work with the people of SLI to investigate the presence of endocrine-disrupting chemicals (EDCs) on the Island. The CBPR team expanded in 2011 to include a research faculty at the University of Alaska, Anchorage. The title of the grant is: “Protecting future generations: assessing and preventing exposures to endocrine-disrupting chemicals on St Lawrence Island.” We are investigating multiple exposure routes to 2 emerging EDCs – polybrominated diphenyl ethers (PBDEs) and perfluorinated compounds (PFCs). The CBPR team is assessing exposures to PBDEs and PFCs in surface waters through analyses of contaminant levels and biomarkers for xenobiotic chemicals in 3-spine stickleback fish. We are analyzing household dust and traditional foods for PBDEs and PFCs. This study includes a human biomonitoring component in order to assess levels of PBDEs and PFCs in human blood serum in relation to measures of thyroid health. We are collaborating with leaders, elders and the youth of SLI to develop measures to prevent and mitigate environmental exposures through community educational programs and public policy actions, which include conducting community-based research institutes for college credit, health fairs for all community members and workshops for health care providers.

### National intervention strategies

In addition to efforts to ensure responsible clean-up of the FUDS, the communities of SLI and ACAT are collaborating on a national level to reform chemicals policy in order to reduce contaminant sources and prevent further contamination of the Arctic. In October 2011, with technical assistance from ACAT, the Native Village of Savoonga introduced a resolution to the Alaska Federation of Natives (AFN), the largest representative annual gathering in the United States of Native peoples (5,000 people attending). AFN voted in support of the resolution entitled “Action for protecting the health of present and future generations by preventing toxic exposures through chemicals policy reform.” The resolution calls on “Alaska senators and representative to the US Congress to take leadership on chemicals policy reform and use the full power of their offices to transform the 35-year-old Toxic Substances Control Act by passing the Safe Chemicals Act.” As a direct result of the AFN resolution and prompting by other Alaska citizens, both Alaska senators agreed to support a bi-partisan safe chemicals bill that was introduced in 2013, national legislation that would reduce production and exposures to persistent, bioaccumulative chemicals and require chemical manufacturers to demonstrate the safety of their products.

### International intervention strategies

Since 2000, the CBPR partners have been working at the international level to support the negotiation and implementation of the Stockholm Convention on Persistent Organic Pollutants (“POPs treaty”), a global legally binding treaty ratified by over 179 nations to eliminate some of the world's most hazardous industrial chemicals. Initially signed in 2001 by more than 100 nations, it bans 12 deadly chemicals worldwide and offers provisions for adding new POPs to the list to be eliminated. The Preamble of the POPs Treaty states “Arctic ecosystems and indigenous communities are particularly at risk because of the biomagnification of persistent organic pollutants and that contamination of traditional food is a public health issue” (29). As a direct result of our technical assistance and formal interventions, 12 additional POPs were added under provisions of the Convention in 2009; in 2011 the pesticide endosulfan was added; and in 2013 the flame-retardant hexabromocyclododecane (HBCD) was also designated for global elimination.

**Fig. 8:**
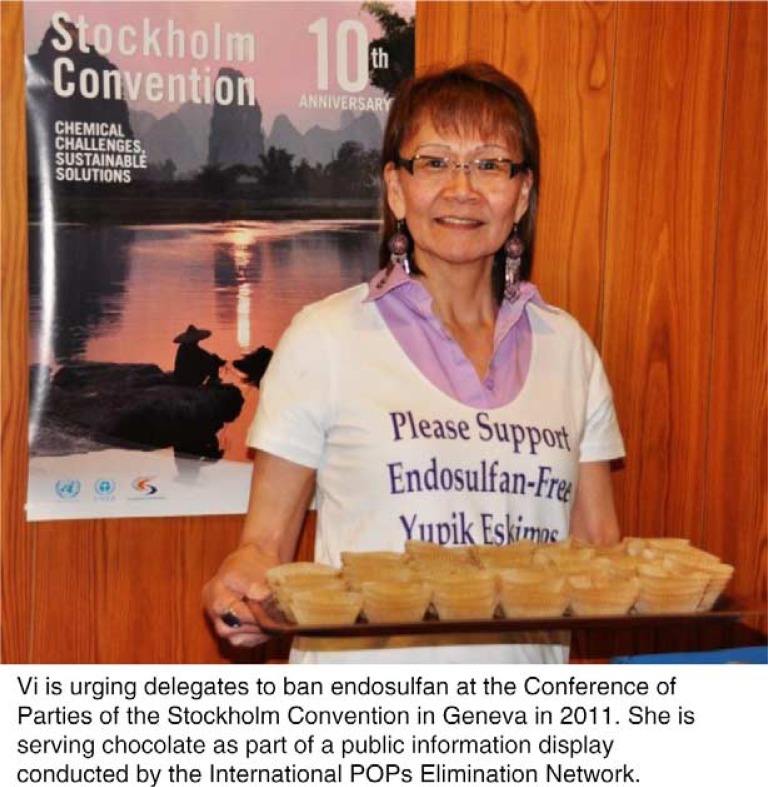
Photo of Viola “Vi” Waghiyi, Yupik from St Lawrence Island and Environmental Health and Justice Program Director, Alaska Community Action on Toxics.

We actively participate in the Global Indigenous Peoples Caucus and the International POPs Elimination Network, a global network of more than 700 public interest groups working together for the elimination of POPs. We provide technical assistance during yearly meetings of scientists who advise the delegates to the POPs treaty, and during biennial meetings of the delegates (see Fig. 8). In 2013, 4 researchers from the SLI CBPR team, 3 of whom are SLI Yupik, participated in the Conference of Parties in Geneva as formal observers, and 2 will go to Rome in October 2013 to participate in the POPs Review Committee. The Stockholm Convention is an important means to decrease the long-range transport of POPs to the Arctic.

In 2013, the World Health Organization (WHO) listed PCBs as carcinogenic “in humans and experimental animals” and “classified PCBs as carcinogenic to humans (Group 1)” (30). David O. Carpenter, MD, a key researcher for our CBPR projects, served as “Invited Specialist” during the decision-making process by the WHO.

## Conclusion

ACAT has been conducting CBPR projects with the people of SLI continuously since 2000. When SLI leaders first came to ACAT in 1997 to request our technical assistance, they asked us to help them determine whether or not their people were being exposed to harmful materials at the 2 abandoned military sites on their Island. Our exposure assessments during the past 13 years demonstrate clearly that the leaders of SLI have reason to be concerned about the health of their people due to presence of carcinogenic chemicals (PCBs) as measured in biomonitoring and environmental samples.

The first CBPR biomonitoring projects conducted in 2000–2003 showed elevated levels of PCBs in blood serum of individuals living in Gambell and Savoonga, but those residents of Savoonga who also hunted, fished, gathered berries, and drank from the Suqi River at the FUDS located at Northeast Cape had even higher levels of PDBs – higher than those living in the rest of the Island who were not associated with Northeast Cape.

The FUDS at Northeast Cape continues to be a source of PCBs, as there are areas in the upstream reaches of the Suqi River's drainage from the FUDS that serve as an on-going source of PCBs to the Suqi watershed. Also, plants from the area near the FUDS have PCB-contaminated dust that should not be ingested.

Beginning in 2003, the Yupik members of the CBPR research team collected cooked or prepared traditional foods from the people on SLI. We determined that elevated concentrations of PCBs in marine mammals that serve as traditional food sources for the people of SLI are primarily derived from long-range transport and represent another major source of PCB exposure.

Beginning in 2011, the CBPR team has been working to determine if EDCs are also affecting the people of SLI.

Since 2000, the CBPR teams have been conducting interventions to address the fact that contaminants from local and distant sources are affecting the lives of the Yupik people who live on SLI. We have been working with policymakers locally, nationally, and internationally to address contaminants affecting the Arctic and to protect future generations.

Based on epidemiological studies of Alaska Native and circumpolar Inuit populations (31–34), it should be noted that other causes of cancer – some of which can be prevented – may be present on SLI such as a high prevalence of tobacco use, aging of the population, and the increasing trend to a Western diet which may also contribute to a large proportion of health issues observed in this community. Our CBPR studies focus entirely on assessing exposures to harmful chemicals; however we believe that future research is needed at SLI to assess disease patterns that might be associated with exposure to the chemicals identified in our exposure assessments.
